# A practical guide on how to handle patients with bleeding events while on oral antithrombotic treatment

**DOI:** 10.1007/s12471-018-1117-1

**Published:** 2018-05-08

**Authors:** M. E. Gimbel, S. C. S. Minderhoud, J. M. ten Berg

**Affiliations:** Department of Cardiology, St. Antonius Hospital, Nieuwegein, The Netherlands

**Keywords:** Anticoagulants, Antithrombotic treatment, Antiplatelet therapy, Haemorrhage, Bleeding

## Abstract

Bleeding is a feared complication in patients who are treated with antithrombotic therapy (oral anticoagulation or antiplatelet therapy). Management of antithrombotic therapy after bleeding poses a dilemma where restarting the crucial medication could lead to recurrent bleeding, while interrupting or even discontinuing treatment could increase the thrombotic risk. In this review, we provide recommendations regarding the treatment of patients with a bleeding event while on oral antithrombotic therapy, based on the literature and expert opinion.

## Introduction

Bleeding is a frequent complication both in patients treated with oral anticoagulation (OAC) and in patients with antiplatelet therapy, in which major bleeding occurs in roughly 5% within one year in both groups [1–8]. Despite the bleeding risk, dual antiplatelet therapy (DAPT), consisting of aspirin with a P2Y12 inhibitor, remains the cornerstone of treatment in patients with acute coronary syndrome (ACS) and those undergoing percutaneous coronary intervention (PCI) with stenting. Moreover, the more potent P2Y12 inhibitors, ticagrelor and prasugrel, are preferred over clopidogrel, further increasing the risk of bleeding [9]. The preferred duration of DAPT after ACS is 12 months, but in patients with a high bleeding risk a shorter duration should be considered, with a minimum of one month in medically managed patients and 6 months in patients who underwent PCI, based on the literature [10, 11]. OAC is indicated in most patients with non-valvular atrial fibrillation (AF). The non-vitamin K oral anticoagulants (NOACs) are recommended in preference to vitamin K antagonists (VKAs) because of the reduced bleeding risk [12]. Major bleeding is associated with a significant increase in the risk of death (11%), myocardial infarction and stroke, possibly because predictors of bleeding have much overlap with predictors of ischaemic events and due to discontinuation of effective antithrombotic drugs when bleeding occurs [13, 14]. Therefore, resuming antithrombotic therapy poses a clinical dilemma: restarting antithrombotic therapy prematurely could lead to recurrent bleeding while delaying reinitiation puts the patient at an increased thrombotic risk.

The purpose of this review is to provide recommendations to enhance clinical decision making regarding the treatment of patients with a bleeding event while on antithrombotic therapy, based on the literature and expert opinion. Our search is shown in the Appendix.

**Figure:**
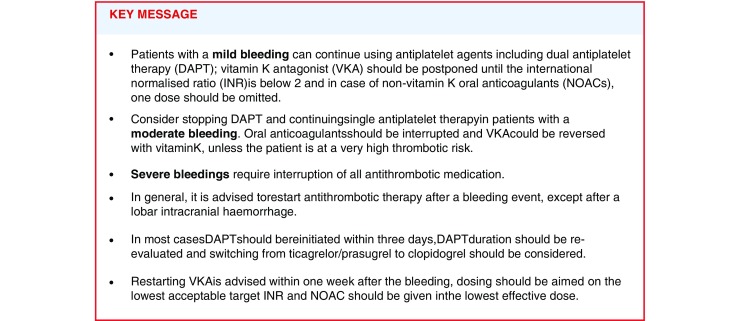

## Recommendations from the guidelines categorised according to severity of bleeding

**Fig. 1:**
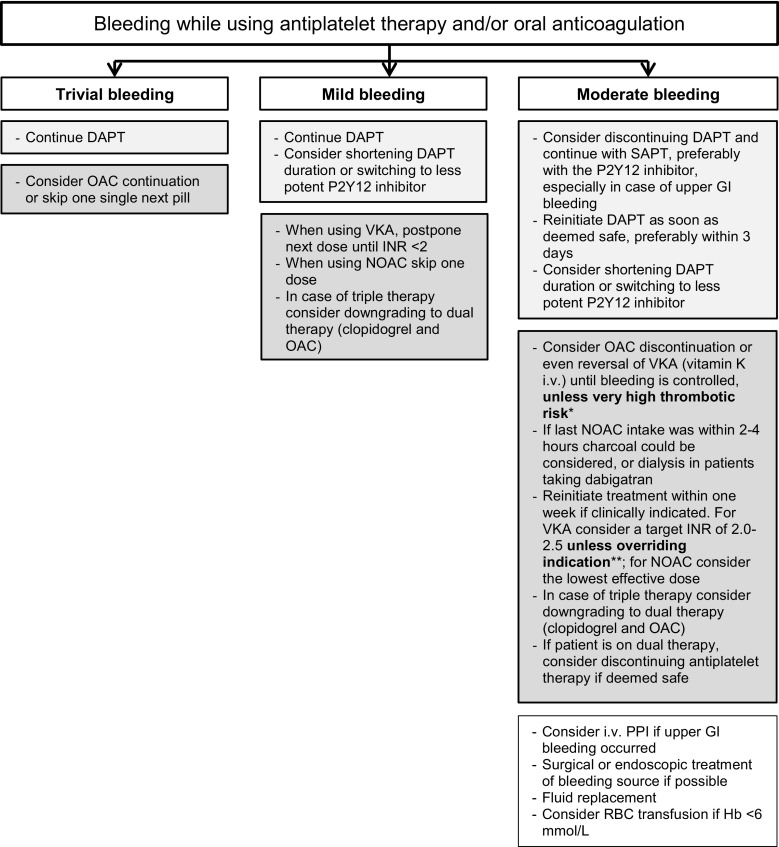
Flow chart with the treatment options in trivial, mild and moderate bleeding events. (*CHA*_*2*_*DS*_*2*_*-VASc* congestive heart failure, hypertension, age ≥75 [doubled], diabetes, prior stroke [doubled]—vascular disease, age 65–74, sex category,* DAPT* dual antiplatelet therapy, *INR* international normalised ratio, *OAC* oral anticoagulation, *VKA* vitamin K antagonist, *NOAC* non-vitamin K oral anticoagulant, *SAPT* single antiplatelet therapy, *PPI* proton pump inhibitor, *GI* gastrointestinal, *RBC* red blood cell, *Hb* haemoglobin, *FFP* fresh frozen plasma, *4F-PCC* four factors prothrombin complex concentrates, *i.* *v*. intravenous. *Mechanical heart valve, cardiac assist device, CHA_2_DS_2_-VASc score ≥4. **Mechanical heart valve, cardiac assist device. ***Mechanical heart valve in mitral position, cardiac assist device)

**Fig. 2:**
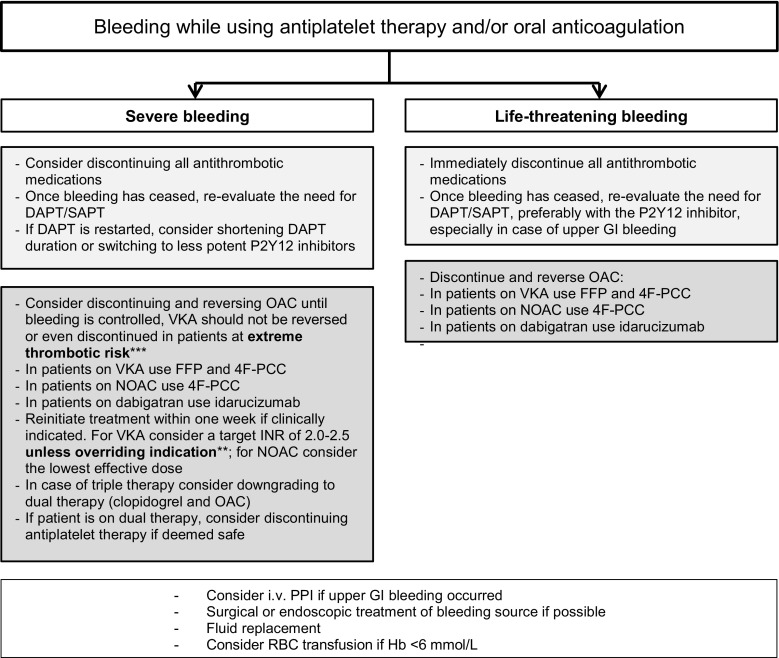
Flow chart with the treatment options in severe and life-threatening bleeding events. (*CHA*_*2*_*DS*_*2*_*-VASc* congestive heart failure, hypertension, age ≥75 [doubled], diabetes, prior stroke [doubled]—vascular disease, age 65–74, sex category,* DAPT* dual antiplatelet therapy, *INR* international normalised ratio, *OAC* oral anticoagulation, *VKA* vitamin K antagonist, *NOAC* non-vitamin K oral anticoagulant, *SAPT* single antiplatelet therapy, *PPI* proton pump inhibitor, *GI* gastrointestinal, *RBC* red blood cell, *Hb* haemoglobin, *FFP* fresh frozen plasma, *4F-PCC* four factors prothrombin complex concentrates, *i.* *v*. intravenous. *Mechanical heart valve, cardiac assist device, CHA_2_DS_2_-VASc score ≥4. **Mechanical heart valve, cardiac assist device. ***Mechanical heart valve in mitral position, cardiac assist device)

First, we will discuss the recommendations from the guidelines regarding the treatment of mild, moderate, severe and life-threatening bleeding, using the same definitions for the bleeding categories. Where possible, we will provide the guideline’s class of recommendation and level of evidence (LoE). Fig. 1 and 2 show flow charts summarising the treatment options stratified by bleeding severity.

### Mild bleeding

The European Society of Cardiology (ESC) DAPT guideline advises in patients with a trivial bleeding not requiring medical attention, to continue antithrombotic treatment without interruption [11]. After a mild bleeding event, defined as needing medical attention without hospital stay, the guideline advises to continue DAPT, however, re-evaluation of DAPT duration and switching from ticagrelor/prasugrel to the weaker clopidogrel should be considered [11]. In case of triple therapy (OAC and DAPT), downgrading to dual therapy (OAC and clopidogrel) should be considered [11]. Patients on VKA with a mild bleeding event are advised to postpone the next dose until an international normalised ratio (INR) <2, patients on NOACs are advised to skip one dose [12, 15].

### Moderate bleeding

A moderate bleeding is a significant blood loss (≥2 mmol/L haemoglobin) or requires hospitalisation, however, the patient is haemodynamically stable [11]. The ESC DAPT guideline recommends interrupting DAPT and switch to single antiplatelet therapy, preferably the P2Y12 inhibitor, especially in upper gastrointestinal bleeding events. Based on limited literature, we advise to restart DAPT within 3 days after the bleeding has been stopped [16, 17]. Furthermore, the guideline advises to consider shortening DAPT duration and switching to the less aggressive P2Y12 inhibitor clopidogrel [11]. Both the ESC DAPT and AF guideline recommend discontinuing OAC, whereby VKA could be reversed, until the bleeding is stopped, unless the patient is at very high thrombotic risk (mechanical heart valve, cardiac assist device, CHA_2_DS_2_-VASc [Congestive heart failure, Hypertension, Age ≥75 [doubled], Diabetes, prior Stroke [doubled]—Vascular disease, Age 65–74, Sex category] score ≥4) [11, 12]. For patients on VKA, vitamin K 5–10 mg intravenously should be considered, bearing in mind it has a slow onset (at least 4–6 hours) [18]. In patients with a NOAC-related bleeding, time is the most important antidote, with a plasma half-life of ~12 hours. Therefore, it is important to inquire about the last dose of NOAC intake and consider factors influencing plasma concentration such as renal function [15, 19]. Charcoal could be administered if the last NOAC intake was within 2–4 hours and dialysis in patients taking dabigatran. A moderate bleeding may require blood transfusions and fluid replacement in addition to specific treatment to stop the bleeding (endoscopic or surgical haemostasis) [12, 19]. According to the guidelines, OAC should be reinitiated within one week, taking into account the lowest acceptable target INR or, in case of NOAC, the lowest effective dose. Patients on triple therapy should be considered for dual therapy with OAC and clopidogrel [11].

### Severe bleeding

The same recommendations as stated under moderate bleeding apply for severe bleeding, defined as a blood loss of >3 mmol/L haemoglobin, requiring hospitalisation in a haemodynamically stable patient, with the addition to consider discontinuing all antithrombotic medication if bleeding persists despite treatment. For patients on antiplatelet therapy, routine thrombocytes infusion seems not to be recommended [18], based on the PATCH trial, randomizing patients with intracerebral haemorrhage to platelet transfusion or not. An increased risk of death or dependence in the group which received platelet transfusion (odds ratio [OR] 1.84) was found [20]. The Dutch guideline for antithrombotic management advises against desamino-D-arginine vasopressin or desmopressin because of the relative contra-indication for patients with coronary artery disease [15, 21].

In patients with a severe bleeding on OAC, stopping and reversing OAC is indicated, unless the patient has an excessive thrombotic risk (mechanical heart valve in mitral position, cardiac assist device) [11]. For VKAs the combination of fresh frozen plasma with four factors prothrombin complex concentrates (4F-PCC; 25–50 U/kg) should be considered (class IIa, LoE C) [9, 12, 21]. Both are faster in antagonising VKA compared with vitamin K [19]. 4F-PCC may also be administered in patients on NOACs when a specific antidote is not available (class IIa, LoE C) [9, 12, 21]. However, PCCs increase the risk of thromboembolic events (>1%) [22, 23]. Patients with a bleeding on dabigatran could be treated with idarucizumab 5 mg intravenously [11, 12, 15, 19] which antagonises dabigatran within 5 minutes, without procoagulant activity [19, 24]. RE-VERSE AD examined the reversal effects of idarucizumab in 301 patients with uncontrolled or life-threatening bleeding, of whom 96% used dabigatran because of AF [24]. The median time to haemostasis after idarucizumab was 2.5 hours in patients without intracranial haemorrhage. Furthermore, antithrombotic treatment (anticoagulant or antiplatelet drugs) was restarted in 73% after a mean of 13 days of which 23% restarted within 3 days [24]. This is in accordance with the DAPT guideline which advises to reinitiate treatment within one week [11].

### Life-threatening bleeding

In a patient with a life-threatening bleeding or bleeding leading to significant disability (e. g. intraocular haemorrhage), all antithrombotic medication should be discontinued immediately. OAC should be stopped and reversed. Afterwards, the need for reinitiating antithrombotic treatment should be evaluated [11, 15, 21]. Based on the very limited literature we advise to restart antithrombotic therapy approximately one month after the bleeding is stopped, except in patients with lobar haemorrhage (*see paragraph intracranial haemorrhage*).

In the following text, the specific situations of restarting medication after gastrointestinal bleeding and intracranial haemorrhage are outlined. The specific advices set out below are in addition to the recommendations stated above. These particular bleeding sites are separately discussed because gastrointestinal bleeding is the most frequently occurring major bleeding event [25–27] and intracranial haemorrhage is the most serious bleeding event, posing enormous difficulties as to whether and when to restart medication.

## Gastrointestinal bleeding when using oral anticoagulation

There are no randomised data on restarting medication after gastrointestinal bleeding. The meta-analysis of Chai-Adisaksopha et al. examined the benefit and risks of resuming anticoagulant therapy following gastrointestinal bleeding [28]. Three studies were selected, including patients using warfarin for various reasons (AF, deep vein thrombosis, pulmonary embolism or prosthetic heart valve), of which 17–83% also used anti-platelet therapy. Resumption of warfarin (in 53% of patients) was associated with a significant reduction in thromboembolic events 9.9% versus 16.4% (hazard ratio [HR] 0.68; 95% confidence interval [CI] 0.52–0.88; *p* = 0.004) and mortality 24.6% versus 39.2% (HR 0.76; 95% CI 0.66–0.88; *p* = 0.0002). However, a numerically increased rate of recurrent gastrointestinal bleeding was observed in patients resuming warfarin (10.1% vs 5.5%, HR 1.20; 95% CI 0.97–1.48; *p* = 0.10). This risk was significantly higher when restarting warfarin within 7 days compared with restarting later [29, 30]. Accordingly, the European Society of Gastrointestinal Endoscopy (ESGE) guideline recommends in patients on (N)OAC with a moderate gastrointestinal bleeding or worse, to stop (N)OAC and restart between 7–15 days after the gastrointestinal bleeding event [17]. Patients with a very high thrombotic risk, e. g. mechanical heart valve, cardiac assist device, CHA_2_DS_2_-VASc score ≥4 may benefit from earlier (first week) resumption [11, 17].

The aforementioned studies all have an observational design and are, therefore, subject to bias through confounding by indication; patients who did not restart OAC had more severe bleeding events and were older compared with restarters.

Specific data regarding restarting NOAC after gastrointestinal bleeding is lacking. Therefore, it seems reasonable to extrapolate data regarding warfarin to the NOACs, keeping in mind the faster therapeutic onset of NOACs compared with warfarin. The four phase III trials comparing the different NOACs with warfarin, showed an increased risk of gastrointestinal bleeding with rivaroxaban, high-dose dabigatran and high-dose edoxaban. Apixaban, low-dose dabigatran and low-dose edoxaban showed comparable gastrointestinal bleeding risk with warfarin [3–6]. Therefore, patients with a gastrointestinal bleeding while using a NOAC should be considered for a lower dose or for apixaban (class IIa, LoE B) [12].

## Gastrointestinal bleeding when using antiplatelet therapy

A double-blinded, randomised controlled trial (RCT) included 156 patients with upper gastrointestinal bleeding on aspirin for secondary prevention and randomised to continuing aspirin or placebo. Recurrent upper gastrointestinal bleeding occurred more frequently in the group treated with aspirin, 10.3% vs. 5.4% (HR 1.9; 95% CI 0.6–6.0). But the number of blood transfusions was equal between both groups, implying relatively mild recurrent bleeding events. Mortality after 8 weeks was, however, significantly lower in patients treated with aspirin, 1.3% vs. 12.9% (HR 0.2; 95% CI 0.06–0.60) [31]. Data regarding gastrointestinal bleeding while on DAPT are scarce.

In accordance, the ESGE guideline and expert consensus paper of the ESC, advises in patients on aspirin or DAPT for secondary prevention with upper gastrointestinal bleeding, to continue aspirin or DAPT if endoscopy shows no active bleeding [16, 17]. Consider a three-day interruption of aspirin in patients with active bleeding by endoscopy (strong recommendation, moderate quality evidence), in case of DAPT, continue the P2Y12 inhibitor and interrupt aspirin for three days [16, 17]. Likewise, the American College of Gastroenterology guideline regarding acute lower gastrointestinal bleeding recommends continuing aspirin for secondary prevention [32]. But, in patients on DAPT, it is advised to interrupt the P2Y12 inhibitor for a maximum of 7 days, aspirin should be continued. However, if the patient suffered from an ACS within 90 days or received a coronary stent within 30 days DAPT should be continued (strong recommendation, low quality evidence) [32]. The ESC DAPT guideline also advises to consider shortening the DAPT duration and switching to DAPT consisting of aspirin with clopidogrel [11].

In addition to the measures mentioned above, the ESGE guideline recommends in patients with upper gastrointestinal bleeding, immediate initiation of high-dose intravenous proton pump inhibitors (PPIs) (strong recommendation, high quality evidence) [17] and to continue infusion until 72 hours post endoscopy [17]. PPIs reduce the risk of upper gastrointestinal bleeding significantly in patients treated with antiplatelet therapy, and numerically in patients treated with OAC. Therefore, it is recommended to continue treatment with oral PPIs after discharge when antiplatelet therapy is reinitiated [11, 16, 33–35], and we advise to consider this when OAC is reinitiated [35].

### Advice

- Stop OAC in patients with a moderate gastrointestinal bleeding or worse [11, 16, 17].
- Restart OAC between 7–15 days after gastrointestinal bleeding [17, 21, 30].
- Patients at very high thrombotic risk, e. g. mechanical heart valve, cardiac assist device, CHA_2_DS_2_-VASc score ≥4 may benefit from resumption in the first week [11, 17].
- Consider low-dose NOAC or apixaban in patients with NOAC-related gastrointestinal bleeding (class IIa, LoE B) [12].
- In patients on aspirin or DAPT for secondary prevention with upper gastrointestinal bleeding, continue aspirin or DAPT if endoscopy shows no active bleeding [16, 17].
- Consider a three-day interruption of aspirin in patients with active bleeding by endoscopy (strong recommendation, moderate quality evidence), in case of DAPT, continue the P2Y12 inhibitor and interrupt aspirin for three days [16, 17].
- Aspirin for secondary prevention should be continued in patients with lower gastrointestinal bleeding.
- In patients on DAPT, the P2Y12 inhibitor should be interrupted for 7 days and aspirin should be continued unless the patient had ACS within 90 days or coronary stent within 30 days (strong recommendation, low quality evidence) [32].
- Consider in patients on DAPT to shorten the duration and to switch to DAPT with aspirin and clopidogrel [16].
- Start with intravenous PPI in case of upper gastrointestinal bleeding and continue with oral PPI after discharge when antiplatelet therapy is reinitiated [11, 17], consider oral PPI when OAC is reinitiated [35].

## Intracranial haemorrhage while using oral anticoagulation

A meta-analysis by Murthy et al. summarised the results of eight retrospective cohort studies, including patients with nontraumatic intracranial haemorrhage (intraparenchymal, subdural and/or subarachnoid haemorrhages) while on OAC [36]. Indication for OAC (mainly VKA) was most often AF, followed by prosthetic heart valve, venous thromboembolism and previous ischaemic stroke; in addition, 5–33% also used antiplatelet therapy. After intracranial haemorrhage, 35.8% resumed VKA, with a mean of one month after intracranial haemorrhage. The results showed a thromboembolic complication rate of 6.7% in patients restarting OAC versus 17.6% in those who did not (rate ratio [RR] 0.34; 95% CI 0.25–0.45), while recurrence of intracranial haemorrhage was numerically higher in patients restarting therapy, 8.7% vs. 7.8% (RR 1.01; 95% CI 0.58–1.77). These data should be interpreted with caution as all studies were subject to confounding by indication restarting VKA in patients with smaller haematomas and a less critical location of bleeding. Data regarding the treatment of intracranial haemorrhage while using NOAC is not available, however, the studies RE-LY, ENGAGE-AF, ARISTOTLE and ROCKET-AF all showed a significantly reduced risk of intracranial haemorrhage with NOACs compared with VKA. Moreover, NOAC-related intracranial haemorrhages were smaller and had a better clinical outcome [3–6]. The ESC AF guideline advises to withhold OAC if bleeding occurred while adequately dosed, in case of uncontrolled hypertension, if the bleeding is located cortically, if there are multiple microbleeds (>10) or if DAPT is needed. Factors supporting reinitiating OAC are: bleeding occurred on VKA or in the setting of overdose; bleeding was traumatic or has a treatable cause; bleeding is located in the basal ganglia; when the white matter lesions are mild; in case of removed subdural haematoma or clipped/coiled aneurysm [12]. Switching to a NOAC after intracranial haemorrhage should be considered (class IIb, LoE B) [12, 15, 16, 37–40]. If it is decided to restart antithrombotic treatment the advice is to start one month after intracranial haemorrhage (class IIb, LoE B) [12, 21, 36].

## Intracranial haemorrhage while using antiplatelet therapy

A meta-analysis including six observational studies, reviewed the treatment decisions made in patients with a primary intracranial haemorrhage while on single antiplatelet therapy [41]. In total, 1916 patients were included of which 825 (43%) resumed antiplatelet therapy. Timing of resumption after intracranial haemorrhage varied widely and data were not always available. In the largest included study (*n* = 759), the median time to restart was 24 days, however, only patients with AF were included [42]. The meta-analysis showed equal recurrence of intracranial haemorrhage or haematoma expansion in both groups [RR 0.84; 95% CI 0.47–1.51; *p* = 0.56], but thromboembolic events were significantly lower in patients restarting antiplatelet therapy [RR 0.61; 95% CI 0.48–0.79; *p* < 0.01].

One study from the meta-analysis included 109 Chinese patients with a first-time aspirin-related primary intracerebral haemorrhage surviving >90 days after admission. All patients used aspirin for secondary prevention. After intracerebral haemorrhage, 19% restarted aspirin after a median of 87 days. Patients who resumed aspirin had less disabilities and a higher rate of ischaemic heart disease (56.8% vs. 23.6%). Importantly, ischaemic events were 2–3 times more common than recurrent intracerebral haemorrhage, which was comparable between both groups. A systolic blood pressure of >140 mm Hg and cerebral amyloid angiopathy were independent predictors of recurrent intracerebral haemorrhage. After intracerebral haemorrhage it takes a few weeks to restore the blood-brain barrier [43], and for the perihaemorrhagic oedema to decrease [44]. Therefore, antiplatelet resumption should be safe after approximately 4 weeks.

Confounding by indication is of particular importance in patients after intracranial haemorrhage because different locations of bleeding are associated with different recurrent rates and disabilities. The most common distinction made in intracerebral haemorrhage is lobar (cerebral cortex and underlying white matter) versus deep (basal ganglia, thalamus, brainstem) with recurrence rates of 15.7% vs. 3.4% (*p* = 0.011) respectively [45].

Intracranial haemorrhage while on DAPT is reported in only 0.3% of patients [7, 8]. We can only speculate on when to restart medication in these patients and presume that DAPT could also be reinitiated after approximately 4 weeks.

### Advice

- Withhold OAC if intracranial haemorrhage occurred while adequately dosed, in case of uncontrolled hypertension, if the bleeding is located cortically, if there are multiple microbleeds (>10) or if DAPT is needed [12].
- Reinitiate OAC if intracranial haemorrhage occurred on VKA or in the setting of overdose, if the bleeding was traumatic or has a treatable cause, if the bleeding is located in the basal ganglia, if the white matter lesions are mild, in case of removed subdural haematoma or clipped/coiled aneurysm [12].
- Switch to a NOAC after intracranial haemorrhage (class IIb, LoE B) [12, 15, 16, 37–40].
- If it is decided to restart antithrombotic treatment, start after one month (class IIb, LoE B) [12, 21, 36].
- Restart single antiplatelet therapy in patients with deep intracranial haemorrhage and a high thrombotic risk after one month [42, 46].
- In patients with lobar intracranial haemorrhage restarting antiplatelet therapy is not advised [45].
- Restart DAPT with aspirin plus clopidogrel, one month after intracranial haemorrhage in ACS patients with DES implantation shorter than 3 months ago and continue DAPT until the minimal advised duration [9].

## Anaemia and recent ischaemic heart disease

The Dutch guideline for blood transfusions advises in patients with a recent (<3 months) myocardial infarction or ongoing ischaemia to consider a liberal blood transfusion regimen, transfusing patients with haemoglobin levels of ≤6 mmol/L [47]. However, the ESC NSTEMI guideline recommends transfusion only in haemoglobin levels <4.5 mmol/L (class IIb, LoE C) [9]. And the American College of Physicians clinical practice guideline and the American College of Cardiology expert consensus document on management of bleeding recommend a target haemoglobin level of >5 mmol/L [48].

The largest RCT addressing restrictive versus liberal transfusion in ischaemic heart disease patients included 838 patients, admitted to the intensive care unit, with haemoglobin levels of 5.5 mmol/L or less, without active bleeding or chronic anaemia [49]. Of these, 257 had ischaemic heart disease, 111 received restrictive and 146 liberal transfusion. In the total study population, a restrictive red-cell transfusion (<4.5 mmol/L) proved non-inferior to a liberal transfusion (<6 mmol/L). However, subgroup analysis of patients with ischaemic heart disease showed a nonsignificant decrease in overall survival in the restrictive transfusion group [50]. The authors conclude that patients with ACS might not benefit from the restrictive transfusion strategy. A smaller RCT, including 110 patients with symptomatic coronary artery disease allocated patients to a restrictive (<5 mmol/L) or liberal (<6 mmol/L) strategy and found also a trend towards fewer major cardiac events and deaths in the liberal transfusion group [51].

### Advice

- Treat anaemic ACS patients according to a more liberal transfusion strategy (<6 mmol/L) [47].

## Conclusion

This article summarises the current literature and recommendations from the guidelines about the management of patients with a bleeding while on antithrombotic therapy. Furthermore, we reviewed and interpreted the data, and based our specific advice to guide the clinician upon this. However, the available guidance is primarily based on observational cohort studies with a high risk of confounding. Overall, it is advised to restart antithrombotic treatment after the bleeding event except for patients suffering from a lobar cerebral haemorrhage. However, the decision to restart antithrombotic therapy should be evaluated per patient and needs multidisciplinary consultation. In addition, we would advise to treat anaemic ACS patients according to a liberal transfusion strategy (<6 mmol/L).

## Appendix

We used references from the ESC and ESGE guidelines and the expert consensus paper of Halversen et al. on management of antithrombotic therapy after bleeding. Furthermore, we hand-searched PubMed using synonyms for bleeding (Hemorrhage[Mesh] OR Hemorrhage[tiab] OR bleeding[tiab]) and antithrombotic treatment (Anticoagulants[Mesh] OR anticoagulant*[ tiab] OR Platelet Aggregation Inhibitors[Mesh] OR antiplatelet*[ tiab] OR antithrombotic[tiab]) and restarting (restart*[ tiab] OR resum*[ tiab] OR reintroduc*[ tiab] OR reinitiat*[tiab]). We also assessed the references from the selected articles.
